# Video Person Re-Identification with Frame Sampling–Random Erasure and Mutual Information–Temporal Weight Aggregation

**DOI:** 10.3390/s22083047

**Published:** 2022-04-15

**Authors:** Jiayue Li, Yan Piao

**Affiliations:** 1 Information and Communication Engineering, Electronics Information Engineering College, Changchun University of Science and Technology, Changchun 130022, China; lijiayue_87@126.com; 2 High-Speed Railway Comprehensive Technical College, Jilin Railway Technology College, Jilin 132299, China

**Keywords:** video person re-identification, mutual information–temporal weight aggregation (MI–TWA), frame sampling–random erasure (FSE), deep learning

## Abstract

Partial occlusion and background clutter in camera video surveillance affect the accuracy of video-based person re-identification (re-ID). To address these problems, we propose a person re-ID method based on random erasure of frame sampling and temporal weight aggregation of mutual information of partial and global features. First, for the case in which the target person is interfered or partially occluded, the frame sampling–random erasure (FSE) method is used for data enhancement to effectively alleviate the occlusion problem, improve the generalization ability of the model, and match persons more accurately. Second, to further improve the re-ID accuracy of video-based persons and learn more discriminative feature representations, we use a ResNet-50 network to extract global and partial features and fuse these features to obtain frame-level features. In the time dimension, based on a mutual information–temporal weight aggregation (MI–TWA) module, the partial features are added according to different weights and the global features are added according to equal weights and connected to output sequence features. The proposed method is extensively experimented on three public video datasets, MARS, DukeMTMC-VideoReID, and PRID-2011; the mean average precision (mAP) values are 82.4%, 94.1%, and 95.3% and Rank-1 values are 86.4%, 94.8%, and 95.2%, respectively.

## 1. Introduction

Person re-ID aims to retrieve the identities of specific persons captured by non-overlapping cameras in different environments [1]. Specifically, given a query image or video of a target person, the goal of person re-ID is to identify this specific person [2,3,4] from a multi-camera web gallery or video library in non-overlapping view domains. With the increased use of surveillance cameras, such as intelligent traffic systems, the wide application of multi-person tracking detection, and the rapid development of intelligent video analysis, the emphasis on public security monitoring and the popularization of intelligent transportation, person re-ID technology has become one of the most important areas in the field of video surveillance.

For image-based person re-ID, extracting effective pedestrian appearance features is an important step. However, the information contained in the image is limited. If the pedestrian information from a camera perspective is seriously missing, it will be difficult to find additional information for identification. Therefore, research on video-based person re-identification is particularly important. The purpose of video-based person re-ID [5,6,7] is to determine whether the person captured by video images from different cameras matches the target person. Compared with image-based person re-ID, more information is available, including information such as pedestrian appearance, pose, and motion. At the same time, it is closer to the actual application scenario because the pedestrians in the monitoring are also set forth in the form of video.

This article focuses on video-based pedestrian re-identification. With the widespread application of deep learning, Recurrent Neural Network (RNN) has also been applied to aggregate image-level features into sequence-level features for video-based person re-identification [8,9,10,11]. Some researchers also use optical flow to build spatiotemporal models with two-stream networks [12]. All these methods significantly improve the performance of video-based person re-identification. However, feature learning of deep learning requires a large number of samples to train the model, so it is easy to overfit on the dataset. Significant appearance variations due to camera viewpoint, background clutter, and especially, partial occlusion [13] can also occur when using this method. The performance of video-based person re-ID usually deteriorates severely under partial occlusion. This problem is difficult to solve because any part of a person may be occluded by other persons and environmental objects, such as bicycles and indicators. Attention mechanisms have been introduced into video-based person re-ID to handle partial occlusion [14]. They select discriminative frames from video sequences and generate informative video representations. While these methods have some tolerance for partial occlusion, discarding occluded frames is not ideal.

To address the occlusion problem of person re-ID, we use an improved ResNet-50 network to extract global and partial features and add frame-sampling–random erasure (FSE) and a mutual information–temporal weight aggregation (MI–TWA) module to obtain a more discriminative person feature representation. Not only can this method improve the training speed of the model, but it can also compensate for the lack of person image data and alleviate the problem of network overfitting.

In summary, our main contributions are as follows:

- A method using FSE is proposed for data enhancement to compensate for the lack of person image data and combine the length of frame sampling time to alleviate the problems of occlusion and noise in video frames.
- MI–TWA module is added to the improved ResNet-50 network [15], which is integrated with global features of equal weight to help reduce the impact of the occluded parts.
- Experimental results obtained using public benchmark datasets show that the proposed method based on FSE and MI–TWA can effectively solve the occlusion problem and improve the accuracy of video-based person re-ID.

## 2. Related Work

### 2.1. Image-Based Person Re-ID

Person re-ID is a challenging task that has been studied for many years. However, it still faces the same problems as other subfields of computer vision, including various poses, lighting, viewpoints, and occlusions. Person re-ID research has mainly focused on two subtasks, namely image-based [3,4,16,17,18,19,20,21] and video-based [22,23,24,25,26] person re-ID. Sun et al. [21] proposed a method that can accurately divide part-level features without resorting to pose estimation, which effectively improves the performance of re-ID. Zhang et al. [23] proposed a dense semantic contrast pedestrian re-identification method, which is also the first method to use fine-grained semantics to solve the problem of pedestrian image misalignment. In our proposed method, the frame-level image features are extracted according to the ResNet-50 and Part-based Convolutional Baseline (PCB) frameworks, respectively, to extract global features and partial features obtained by horizontal partitioning, so that they can complete subsequent temporal aggregation.

### 2.2. Video-Based Person Re-ID

Compared with image-based person re-ID, the samples in video-based person re-ID contain more frames and additional temporal information. Therefore, some existing methods have attempted to model additional temporal information to enhance video representations, and other methods have used 3D-CNNs [27,28] to explore spatiotemporal cues. For example, for spatial information, Li et al. [23] attempted to extract aligned spatial features across multiple images through different spatial attention modules, and Yang et al. [29] proposed a spatial-temporal GCN (STGCN) for mining spatial and temporal relations. 

Another class of methods [8,30] has utilized the additional information of optical flow and adopted a two-stream structure [31] for discriminative feature learning. However, optical flow only represents the local dynamics of adjacent frames and may introduce noise because of spatial asymmetry. The VRSTC [25] framework proposed by Hou et al. applied a generative adversarial network approach to the study of video-based person re-ID for the first time and recovered occluded local areas through upper and lower frame information. Using de-occluded video for feature learning can effectively solve occlusion problems. The random erasure method proposed by Zheng et al. [32] has also been applied in person re-ID. In person images, the problem of inaccurate matching caused by partial occlusion or incompleteness is solved by randomly selecting a rectangular area and erasing it. Unlike the previous methods, in this paper, we perform frame sampling-based random erasure in the image sequence input stage to reduce overfitting, improve the network generalization ability, and solve the occlusion problem in person re-ID. We strive to introduce the concept of mutual information into the temporal weight aggregation module and aggregate the extracted frame-level global and local features by assigning temporal weights according to the entropy value of mutual information to obtain better spatiotemporal features and reduce noise interference.

## 3. Methodology

### 3.1. Person Re-ID Network Based on Frame Sampling–Random Erasure and Mutual Information–Temporal Weight Aggregation

**Figure 1:**
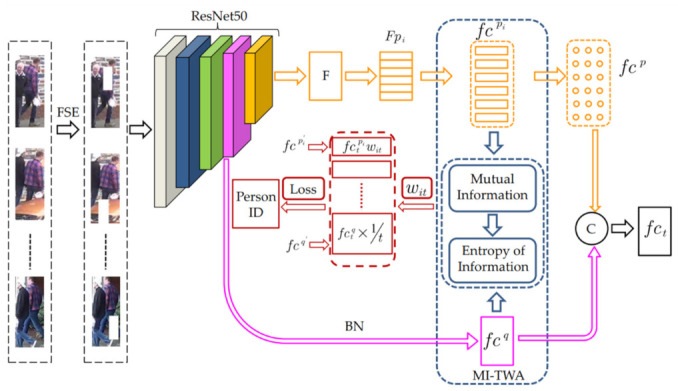
Architecture of our method. The FSE-processed video sequence is used as the input of the network. The ResNet-50 is used to extract the global feature $fc^{q}$ and the partial feature $fc^{p}$. $fc^{q}$ and $fc^{p}$ are fused to obtain the frame-level image feature $fc_{t}$. $fc^{p_{i}}$ and $fc^{q}$ are aggregated by the temporal weights. The temporal weight $W_{it}$ of $fc^{p_{i}}$ is calculated by mutual information entropy value. The temporal weight of $fc^{q}$ is calculated by equal weighting. $fc^{p_{i}^{\prime }}$ and $fc^{q^{\prime }}$ are concatenated as the final feature representation of this sequence. By combining $L_{triplet}$ and $L_{soft\max}$, the network model is trained.

The overall structure of the proposed method is shown in Figure 1. First, the FSE operation is performed on the input video sequence, and the network model is used for feature extraction. The network model structure consists of a backbone network and three branch networks, namely, global feature branch, partial feature branch, and mutual information–temporal aggregation branch. 

The backbone network uses ResNet-50 CNN and the last pooling layer and the fully connected layer of the network are removed. The global feature branch first extracts the features output by the fourth convolution block conv_4 in the ResNet-50 network and performs dimensional upgrade processing through 1 × 1 convolution. In order to preserve the differences in the characteristics of different pedestrians, BN (Batch Normalization) [33] is used for normalization to obtain the global feature $fc^{q}$. For the partial feature branch, we denote the feature map extracted from conv_4 and conv_5 of the ResNet-50 backbone as *F*. *F* is horizontally partitioned into *p* parts, denoted as *Fpi*, *i* = 1,…, *N* [34]. To prevent overfitting through a bottleneck [15], each part of the feature map *Fpi* is processed by average pooling and 1 × 1 convolutional layers, obtaining a partial feature vector $fc^{p_{i}}$. Each partial feature $fc^{p_{i}}$ is fed into a corresponding fully connected layer and a softmax layer is employed to predict the ID of each input image, denoted as $fc^{p}$.

**Figure 2:**
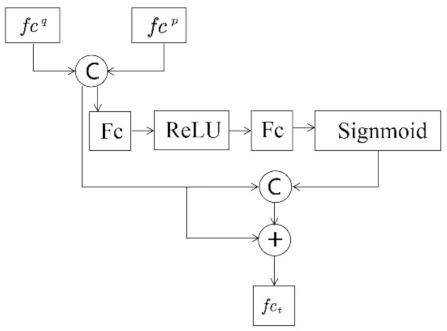
Feature fusion network.

Based on the feature map channel attention mechanism, we designed a feature fusion network [15]. The network structure is shown in Figure 2. The global and partial features are inputs. After cascading, the bottleneck mechanism is used to build a channel attention mechanism to promote interactions between different features and information exchange. At the same time, the residual structure is used to accelerate and stabilize convergence so that, finally, the frame-level feature $fc_{t}$ is obtained.

The mutual information–temporal aggregation branch combines global features with equal weights, aggregates partial features with different time weights according to the entropy value of mutual information, and obtains sequence features by connecting the weighted global features to the partial features; finally, the triple loss function [35] and the softmax loss function [27,36] are combined to train the entire network.

### 3.2. Data Enhancement of Frame Sampling–Random Erasure

As the length of the video sequence varies greatly in the model, the visual information of the entire video is used to solve this problem. For the input video, the entire video is evenly divided into T-consecutive person trajectory sequences. During training, each person’s trajectory sequence is randomly sampled in any frame, and then a sampled frame with some temporal information is taken as input.

**Figure 3:**
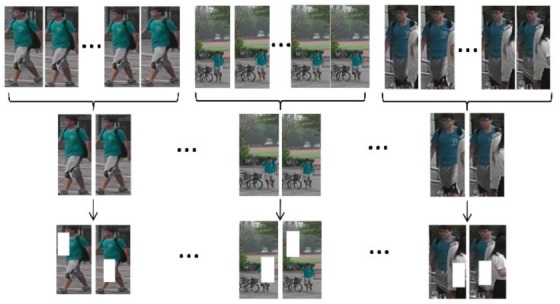
Pipeline of frame sampling–random erasure.

For the situation where the target pedestrian is disturbed or partially occluded, to solve the occlusion problem and improve the generalization ability of the model, random erasure is performed after frame sampling to occlude parts of the image at random positions. This method can generate enhanced images with various degrees of occlusion, making the model more robust to noise and occlusion. For a video sampling frame in a batch, we randomly selected a rectangular area $f_{e}$ from the image frame S=W×H for pixel erasure. The height and width ratios of the random erasure area are $r_{h}$ and $r_{W}$, respectively. The random erase area is $f_{e}=W_{h}\times H_{e}$, $W_{e}=W\times r_{W}$, and $H_{e}=H\times r_{h}$. A point $P\left(x_{e},y_{e}\right)$ is randomly selected in the image as $x_{e}+W_{e}\leq W$ and $y_{e}+H_{e}\leq H$,then the area $I_{e}=\left(x_{e},y_{e},x_{e}+W_{e},y_{e}+H_{e}\right)$ is selected as the random rectangular area to be erased. Otherwise, we continue searching for the point *P* until the required area $I_{e}$ is selected, as well as training images with different degrees of occlusion are generated to enhance the data. Detailed flow of the FSE data augmentation method is shown in Figure 3.

### 3.3. Mutual Information–Temporal Weight Aggregation

Next, we need to merge the frame-level features from the sampled sequence in the temporal dimension. Because of changes in human poses, occlusion, and viewpoint, not all frames have the same amount of information. We are more concerned with frames that provide explicit person feature information, so we calculate the weight $W_{it}$ of the *t*-th frame in the *i*-th part by the entropy value of the mutual information.

Our method sets the local feature horizontal part *N, N = 6*. Useful information related to the non-random erasure part in the partial feature and the person in the global feature is defined as mutual information MI (Mutual Information), which is denoted as follows:

(1) $$MI_{i}\left(fc\right)=p\left(fc^{q}\right)\log\frac{p\left(fc^{p_{i}},fc^{q}\right)}{p\left(fc^{p_{i}}\right)p\left(fc^{q}\right)},i=1,\ldots ,N,$$

where $p\left(fc^{p_{i}}\right)$ denotes the probability that a local feature appears in the entire global feature; $p\left(fc^{q}\right)$ denotes the probability of the person part in the entire global feature; $p\left(fc^{p_{i}},fc^{q}\right)$ denotes the ratio of the number of the person part in the global feature that contains partial features and the number of the entire global feature. 

The entropy value of the amount of information is denoted as follows:

(2) $$E\left(fc^{p_{i}}\right)=MI_{i}\left(fc\right)\sum_{i=1}^{N}p\left(fc^{p_{i}}\right)\log\left(p\left(fc^{p_{i}}\right)\right),$$

then, the mutual information entropy value of the *t* frame is normalized to obtain the time weight as follows:

(3) $$W_{it}=\frac{1-E\left(fc_{t}^{p}\right)}{\sum_{i=1}^{N}\left[1-E\left(fc_{t}^{p_{i}}\right)\right]},$$

the partial features are aggregated with and temporal weights , which are calculated as follows:

(4) $$fc^{p_{i}^{\prime }}=\sum_{t=1}^{T}W_{it}fc_{t}^{p_{i}},$$

the global features are merged with equal weights *1/t*, which is calculated as follows:

(5) $$fc^{q^{\prime }}=\sum_{t=1}^{T}\frac{1}{t}fc_{t}^{q}.$$

Finally, the partial features $fc^{p_{i}^{\prime }}$ and global features $fc^{q^{\prime }}$ are connected to represent sequence features. In the train stage, combination of cross entropy loss and batch triplet loss was used to train the network. In the test stage, Euclidean distance of the connected features for *L2* normalization was used to evaluate the similarity of the video sequences.

### 3.4. Loss Function

The same loss function in the global feature branch and the partial feature branch, $L_{triplet}$ [35] and $L_{soft\max}$ [27,36], are set. $L_{soft\max}$ is denoted as follows:

(6) $$L_{soft\max}=-\frac{1}{PK}\sum_{i=1}^{P}\sum_{n=1}^{K}p_{i,n}\mathrm{lg}q_{i,n},$$

where $p_{i,n}$ and $q_{i,n}$ are the true and predicted identities of the samples.

(7) $$L_{triplet}=-{\sum_{i=1}^{P}\sum_{a=1}^{K}\left[\alpha +\underset{p=1\cdots K}{\max}{\left\|f_{a}^{\left(i\right)}-f_{p}^{\left(i\right)}\right\|}_{2}-\underset{\begin{array}{c} n=1\cdots K\\ j=1\cdots P\\ j\neq i \end{array}}{\min}{\left\|f_{a}^{\left(i\right)}-f_{n}^{\left(j\right)}\right\|}_{2}\right]}_{+},$$

In Formula (7), $f_{a}$, $f_{p}$, $f_{n}$ are features extracted from target samples, positive samples, and negative samples. Positive samples are persons with the same identity as the target sample; negative samples are persons with different identities from the target samples; α is the hyperparameter used to control the distance within the sample; *P* is the number of pedestrians extracted when training each batch; *K* corresponds to the number of images extracted for each person during training. By combining $L_{triplet}$ and $L_{soft\max}$, the network model is optimized, guiding the network to explore according to the features of the discriminative power; *L* is denoted as follows:

(8) $$L=L_{triplet}+L_{soft\max}.$$

## 4. Experiments

The experimental environment in this paper is based on Ubuntu 20.04, Cuda10.2 and Pytorch 1.8.1, and torchvision 0.9.0 deep-learning framework. The hardware configuration includes three GPUs, GTX 3070Ti (8 GB video memory), and the programming language Python 3.8. To demonstrate the effectiveness of our method on the problem of person re-ID, we evaluate it on three benchmark datasets—MARS, DukeMTMC-VideoReID, and PRID-2011, and compare it with state-of-the-art methods.

### 4.1. Datasets and Evaluation Protocols

**Datasets**. MARS [37] is one of the largest video person re-ID datasets to date. The MARS dataset includes 1261 persons captured by six cameras and each person has at least one sequence of person trajectories from two cameras. All bounding boxes and trajectories of MARS are automatically generated, so they are large in scale and difficult to match at the same time, which has the characteristics of false detection interference.

The PRID-2011 [38] dataset provides video clips of multiple people under two different static surveillance cameras. Cameras A and B have 385 people and 749 people, respectively, and 200 people appear in both the A and B perspectives. The length of the video sequences varies from 5 to 675 frames, with an average of 100 frames. The dataset is collected in uncrowded outdoor scenes with relatively simple and clean backgrounds with little occlusion.

The DukeMTMC-VideoReID [39] dataset is a subset of the DukeMTMC dataset for video-based person re-ID. It consists of images collected by eight cameras with different viewing angles at the Duke University campus, with 36,441 manually detected images of 1812 persons. Because the dataset is manually annotated, for each identity mark there is only one video clip. The dataset includes 702 identities for training, 702 identities for testing, and 408 identities as distractors. Table 1 presents the statistics of the three video datasets.

**Evaluation Metrics**. The metrics of cumulative matching characteristic (CMC) and mean average precision (mAP) are used for evaluation [23,37,40,41]. CMC is based on the retrieval capability of the algorithm to find the correct identity within the top-k ranked matches. We report Rank-1, Rank-5, and Rank-10 CMC accuracies. The mAP metric is used to evaluate algorithms in multi-shot re-identification settings where multiple instances of the same identities are present in the gallery.

**Experimental parameter settings**. This experiment uses ResNet-50 pre-trained on ImageNet as the backbone network. For network training, we use the Adam optimizer, which iteratively updates the neural network weights based on the training data. First, we randomly select *T* = 8 frames from the input video sequence and then randomly select *P* = 8 identities to sample each mini batch. We randomly sample *K* = 4 videos for each identity from the training set sequence and then perform random erasure, setting the erasure height ratio to 1/3. We also resize images to 128 × 256. To make the objective function converge and optimize the network model, the initial value of the learning rate is set to 0.01, and it is reduced to 1/10 of the previous value every 20 cycles until the learning rate reaches 0.0001. The margin parameter of the triple loss function is set to 0.3.

### 4.2. Experimental Evaluation

#### 4.2.1. FSE and MI–TWA Partial Features Improve Performance

To verify the effectiveness of the proposed method, three datasets of video person re-ID are tested and analyzed. The experimental results obtained by using the baseline method, the baseline + FSE method, and the baseline + MI–TWA are presented in Table 2.

The baseline method uses ResNet-50 as the backbone network. After global average pooling, the feature vector of the video segment dimension is 2048 and it only contains the basic network structure of the global feature branch and the softmax cross-entropy loss function.

From Table 2, we observe the following effects of adding FSE to the baseline method: On the MARS dataset, Rank-1 increases from 80.2% to 82.8% and mAP increases from 73.8% to 76.3%. On the DukeMTMC-VideoReID dataset, Rank-1 increases from 86.8% to 90.6% and mAP increases from 84.2% to 88.4%. On the PRID-2011 dataset, Rank-1 increases from 85.1% to 87.3% and mAP increases from 86.3% to 89.2%. These results show that FSE can prevent overfitting and effectively improve recognition performance.

The effect of adding partial feature temporal weight aggregation using mutual information to the baseline method is as follows: On the MARS dataset, Rank-1 increases from 80.2% to 84.5% and mAP increases from 73.8% to 80.8%. On the DukeMTMC-VideoReID dataset, Rank-1 increases from 86.8% to 92.6% and mAP increases from 84.2% to 92.2%. On the PRID-2011 dataset, Rank-1 increases from 85.1% to 90.7% and mAP increases from 86.3% to 92.5%. The experimental results show that in the case of changes and occlusions, partial feature temporal weight aggregation can effectively refine temporal features and increase the distinguishability of feature representation by setting different temporal weights according to the different amounts of information provided by the partial features of all frames.

#### 4.2.2. Performance Improvement of Partial Feature Method with Joint FSE and MI–TWA

From Table 3, we observe that when the FSE method is used with Baseline + MI–TWA, the accuracy improves significantly. Baseline + MI–TWA+FSE outperforms Baseline + MI–TWA by 4.5% (95.2%/90.7%) Rank-1, 2.2% (94.8%/92.6%) Rank-1, and 1.9% (86.4%/84.5%) Rank-1, respectively, and by 2.8% (95.3%/92.5%) mAP, 1.9% (94.1%/92.2%) mAP, and 1.6% (82.4%/80.8%) mAP, respectively, on the PRID-2011, DukeMTMC-VideoReID, and MARS datasets. The experimental results fully demonstrate that the proposed method can effectively improve the performance of person re-ID.

#### 4.2.3. Influence of Video Sequence Length T and Random Erasure Height Ratio on Performance

Table 4 presents the results of comparing the performances of video sequences with different lengths. Besides changing the length *T* of the video sequence, other parameters remain unchanged. *T* = 1 is a model for a single image that does not use temporal features. From Table 4, we observe that an increase in the sequence length *T* results in an improvement in mAP and rank accuracy scores. Compared with *T* = 4, *T* = 8 results in a slight change in both mAP and rank indicators. However, both *T* values have high indicators. Therefore, choosing *T* = 4 in the experiment can provide good results.

From Table 5, we observe that when the video length is *T* = 4, different erasure height ratios have different effects on the network performance. When the ratio is 1/2, the network convergence is relatively poor and the index is relatively low. When the ratio is 1/3, the network has a certain advantage in mAP and the Rank-1, Rank-5, and Rank-10 values achieve the best results. However, when the ratio is 1/2, the erasure area is too large, obscuring a significant amount of information with discriminative rows, thus, resulting in poor network performance.

### 4.3. Comparison with State-of-the-Art Methods

We compare the proposed method with state-of-the-art video-based re-ID methods, including MARS [37], Temporal Attention Model + Spatial Recurrent Model (TAM + SRM), Sequential Decision Making (SDM), VRSTC, GLTR, Attribute-Driven Feature Disentangling and Temporal Aggregation (ADFD-TA), and other methods. The results are summarized in Table 6.

From Table 6, on the PRID-2011 small video dataset, our method can achieve 95.2% in Rank-1 and 95.3% in mAP. On the newer video dataset, DukeMTMC-VideoReID, our method can achieve 94.8% in Rank-1 and 94.1% in mAP. On the more challenging video dataset, MARS, our method can achieve 86.4% in Rank-1 and 82.4% in mAP. Compared with GLTR [33] and VRSTC [25], our method is lower in Rank-1, but higher in mAP than GLTR [33] and VRSTC [25]; Rank-1 has a series of random factors, while mAP is a more comprehensive evaluation metric, which can indicate better performance. We observe that the method of joint FSE and MI–TWA proposed in this paper is better than the current state-of-the-art methods in comprehensive performance. The proposed method effectively enriches person feature information and further improves the video person re-ID recognition rate.

To further verify the generalization ability of the network model, cross-domain testing experiments are performed. The results are shown in Table 7. In Table 7, M and D represent MARS and DukeMTMC-VideoReID, respectively; the letters on the left and right of the arrow (‘’→’’) represent the training set and the test set, respectively. We find that Rank-1 and mAP of the proposed model can only reach 58.6% and 35.7%. Analysis of the results shows that the training set and test set in the person re-ID dataset are disjoint and the features of each dataset are quite different. For example, in the MARS dataset, persons mostly wear summer clothes, such as short sleeves and shorts; however, the pedestrians in DukeMTMC-VideoReID are wearing jackets and trousers and the colors are relatively dull, thus, leading to poor cross-domain performance of the model. The experimental results show that the generalization ability of the proposed model has been improved to a certain extent, but the overall cross-domain recognition ability needs to be further improved.

## 5. Conclusions

In this paper, the temporal modelling method based on video person re-ID is improved and the weight distribution of the partial feature information entropy values on the temporal information of persons is refined, which significantly improves the accuracy of video-based person re-ID. Moreover, frame sampling is adopted. A data augmentation method of random erasure enriches the representation of video person features. We conduct evaluation experiments on the small PRID-2011 dataset and the large DukeMTMC-VideoReID video dataset. A series of comparative experiments are also performed on the large, highly representative MARS video dataset. Experimental results on the three video datasets show that the proposed method of joint FSE and MI–TWA can effectively extract discriminative person feature representations, solve person occlusion problems, and achieve accurate person recognition. It is superior to many existing video-based person re-ID methods in terms of degree and efficiency; however, its cross-domain recognition rate needs to be improved. The main task of future work is to improve the recognition rate of cross-domain testing and combine the proposed method with a target detection or tracking algorithm and apply it to the actual multi-camera monitoring environment to achieve accurate recognition and continuous and stable tracking of target persons.

## Figures and Tables

**Table 1:** Statistics of video datasets.

Dataset	#ID	#Seq	#Train	#Test	#Cam	#Label
PRID-2011	200	400	89	89	2	Manual annotation
MARS	1261	20478	625	636	6	Deformable Part Models (DPM)
DukeMTMC-VideoReID	1812	4832	702	702	8	Manual annotation

**Table 2:** Performance comparison of various components in the proposed method. The Rank-i CMC accuracies and mAP scores are reported.

MARS					DukeMTMC-VideoReID				PRID-2011			
	mAP	Rank-1	Rank-5	Rank-10	mAP	Rank-1	Rank-5	Rank-10	mAP	Rank-1	Rank-5	Rank-10
Baseline	73.8	80.2	89.1	92.3	84.2	86.8	88.2	91.9	86.3	85.1	91.7	95.2
Baseline + FSE	76.3	82.8	90.5	92.9	88.4	90.6	91.6	93.3	89.2	87.3	95.3	97.6
Baseline + MI–TWA	80.8	84.5	91.6	94.2	92.2	92.6	95.5	96.3	92.5	90.7	97.2	98.2

**Table 3:** Comparison between Baseline + MI–TWA+FSE and Baseline + MI–TWA models. The Rank-i CMC accuracies and mAP scores are reported.

	MI–TWA			MI–TWA + FSE		
Dataset	PRID-2011	DukeMTMC-VideoReID	MARS	PRID-2011	DukeMTMC-VideoReID	MARS
Rank-1	90.7	92.6	84.5	95.2	94.8	86.4
Rank-5	97.2	95.5	91.6	97.3	96.1	92.3
Rank-10	98.2	96.3	94.2	98.5	96.6	94.7
mAP	92.5	92.2	80.8	95.3	94.1	82.4

**Table 4:** Performance comparison of different video sequence lengths.

PRID-2011					MARS				DukeMTMC-Video ReID			
T	Rank-1	Rank-5	Rank-10	mAP	Rank-1	Rank-5	Rank-10	mAP	Rank-1	Rank-5	Rank-10	mAP
1	88.7	89.1	92.3	83.7	79.0	86.9	90.2	71.1	81.7	86.6	89.6	81.2
2	92.3	94.8	96.4	91.8	82.9	90.1	92.4	76.7	88.2	92.6	94.7	88.5
**4**	**95.2**	**97.3**	**98.5**	**95.3**	**86.4**	**92.3**	**94.7**	**82.4**	**94.8**	**96.1**	**96.6**	**94.1**
8	95.0	97.2	98.2	95.0	86	92.2	94.5	80.3	92.6	96.0	96.3	92.8

**Table 5:** Comparison of the impact of different erasure height ratios on network performance.

MARS					DukeMTMC-VideoReID			
Ratio	Rank-1	Rank-5	Rank-10	mAP	Rank-1	Rank-5	Rank-10	mAP
1/2	82.3	91.4	93.8	75.6	88.7	93.8	94.7	89.2
**1/3**	**86.4**	**92.3**	**94.7**	**82.4**	**94.8**	**96.1**	**96.6**	**94.1**

**Table 6:** Comparisons of our proposed approach to the state-of-the-art methods.

Method	MARS		DukeMTMC-VideoReID		PRID-2011	
	Rank-1	mAP	Rank-1	mAP	Rank-1	mAP
MARS [37]	68.3	49.3	-	-	77.3	-
TAM + SRM [9]	70.6	50.7	-	-	79.4	94.4
SDM [42]	71.2	-	-	-	85.2	-
ADFD-TA [43]	82.6	71.2	-	-	93.9	-
GLTR [33]	87.0	78.5	96.3	93.7	95.5	-
VRSTC [25]	88.5	82.3	95.0	93.5	-	-
**MI–TWA + FSE(Ours)**	**86.4**	**82.4**	**94.8**	**94.1**	**95.2**	**95.3**

**Table 7:** Performance of our models on the cross-domain condition.

Model	M→D		D→M	
	Rank-1 mAP		Rank-1 mAP	
MI–TWA + FSE	48.2	31.5	58.6	35.7

## Data Availability

Not applicable.
